# Evaluation of Metabolite Profiles of Ginseng Berry Pomace Obtained after Different Pressure Treatments and Their Correlation with the Antioxidant Activity

**DOI:** 10.3390/molecules26020284

**Published:** 2021-01-08

**Authors:** Se Rin Choi, Mee Youn Lee, Chagam Koteswara Reddy, Sang Jun Lee, Choong Hwan Lee

**Affiliations:** 1 Department of Bioscience and Biotechnology, Konkuk University, Seoul 05029, Korea; csr0701@gmail.com (S.R.C.); kkamlice@hanmail.net (M.Y.L.); koteswarreddychagam@gmail.com (C.K.R.); 2 Research Institute for Bioactive-Metabolome Network, Konkuk University, Seoul 05029, Korea; 3 Holistic Bio Co., Gyeonggi-do, Seongnam 13494, Korea; leesjun2006@gmail.com

**Keywords:** ginseng berry pomace, metabolite profiling, ultrahigh-pressure treatment, solvent-solvent extraction, antioxidant activity

## Abstract

Ginseng berry pomace (GBP) is a byproduct of ginseng berry processing and is rich in numerous bioactive components, including ginsenosides and their derivatives. The application of GBP as a beneficial biomaterial is currently limited. In this study, we aimed to evaluate their potential as a promising source of bioactive compounds using metabolite profiling. The GBP obtained after different ultra-high-pressure (UHP) treatments was analyzed by GC-TOF-MS and UHPLC-LTQ-Orbitrap-MS/MS. In multivariate analyses, we observed a clear demarcation between the control and UHP-treated groups. The results demonstrated that the relative abundance of primary metabolites and a few ginsenosides was higher in the control, whereas UHP treatment contained higher levels of fatty acids and sugars. Furthermore, GBPs were fractionated using different solvents, followed by UHPLC-LTQ-Orbitrap-MS/MS analyses. The heatmap revealed that phenolics (e.g., quercetin, kaempferol) and fewer polar ginsenosides (e.g., F4, Rh2) were abundant in the ethyl acetate fraction, whereas the levels of lignans (e.g., 7-hydroxysecoisolariciresinol, syringaresinol) and fatty acids (e.g., trihydroxy-octadecenoic acid, oxo-dihydroxy-octadecenoic acid) were high in chloroform. Correlation analysis showed that phenolics, less polar ginsenosides, and fatty acids were positively correlated with the antioxidant activity of GBP. Our study highlights GBP as a functional ingredient for the development of high-quality ginseng berry products.

## 1. Introduction

Ginseng (*Panax ginseng* C.A. Meyer) is a perennial herb belonging to the Araliaceae family that is widely used as a traditional herbal medicine worldwide. Ginseng contains abundant bioactive compounds, including ginsenosides, polysaccharides, fatty acids, and mineral oils [1]. Ginseng roots are rich in ginsenosides and saponins, which contribute to its numerous biological activities, including anti-diabetic, anti-carcinogenic, anti-stress, and anti-aging [2,3]. Several of these bioactivities are associated with ginsenosides and their derivatives. In ginseng, the compound ginsenosides are distributed in different plant parts, including the roots, leaves, and berries. However, most ginseng studies have primarily focused on ginsenosides extracted from ginseng roots [4,5]. In recent years, several studies have reported that the ginseng berry (GB) has several health benefits, such as antioxidant, anti-cancer, and anti-aging effects [2,3]. Additionally, GBs contain higher levels of total ginsenosides compared to those in ginseng roots and exhibit different profiles of ginsenosides [6]. Furthermore, syringaresinol, the lignan compound, which is the key factor in melanogenesis inhibition, was only found in GBs [7]. Although GBs have numerous health benefits, they are not commercially used and are often discarded as a “useless by-product” [8]. Recently, for the development of new cosmetic products and beverages fortified with functional food ingredients, the food and pharmaceutical/cosmetic industries have focused on the potential of GBs as a beneficial biomaterial.

Usually, in food industries, the ultra-high-pressure (UHP) process is used to enhance the levels of crude saponins and ginsenosides in ginseng extracts [9]. UHP processing is a food preservation technique in which high pressure (50–1000 MPa) is applied to foods, including milk, fruit, and vegetables, without heating [4]. It is useful for increasing the shelf life of products and for enhancing the safety, quality, and extraction yields of food products [10]. However, after UHP treatment, a considerable amount of waste, also known as pomace, is generated and disposed of, which causes environmental issues. To overcome these drawbacks, some industries re-use it as a functional ingredient, but in limited areas, such as fertilizer and livestock feed [11].

Pomace is a byproduct produced by the fruit- and vegetable-processing industries that contains pulp, peel, and seeds. Fruit pomace contains numerous bioactive compounds and is used as a natural food additive. Recently, efforts have been made to utilize fruit industrial waste, and studies have already reported the utilization of pomace from mango, watermelon, grapes, tomatoes, and carrots, among others, for the production of bioactive compounds [12,13,14,15]. However, there have been no reports on the utilization of pomace from GBs (GBP) for its bioactive compounds’ potential as functional ingredients. Therefore, it would be beneficial to investigate its potential and alternative uses to reuse waste for mitigation of environmental issues. Moreover, to overcome these drawbacks and to reduce environmental issues, many researchers have explored optimal extraction techniques for isolation of functional metabolites of fruit pomace [16,17].

Metabolomics has been used in recent years as an effective technique in a variety of research areas, including food science, agriculture, and microbiology [18]. Plant metabolites play an essential role in growth and development under stress through maintenance of cell integrity and the induction of signaling, energy storage, membrane formation and scaffolding, and through whole-plant resource allocation [19]. Since the various metabolites render plant-based foods and food additives highly amenable in the search for metabolic markers of food authenticity or nutritional quality, metabolomic approaches have become vital for food authentication and the analysis of food adulteration [20]. Recently, several studies have been performed to analyze the functional metabolites of fruit pomace by mass spectrometry (MS)-based metabolite profiling [21,22,23]. However, there have been limited reports on the metabolic profile of GBP, the solid remains that are extracted from GB industries after UHP treatment. Therefore, we used metabolite profiling based on MS to investigate the potential of GBP.

The present study aimed (1) to investigate the relative contents of significantly different metabolites obtained after different processing treatments of GBP, and (2) to explore the antioxidant activity and contents of functional metabolites in solvent fractions of GBP. Based on findings of the present study, we believe that GBP can be used as a source of bioactive compounds and we suggest an alternative method of replacing artificial food additives with natural compounds present in GBP.

## 2. Results

### 2.1. Comparative Evaluation of Different UHP-Treated GBP Samples Based on Metabolite Profiles

**Figure 1:**
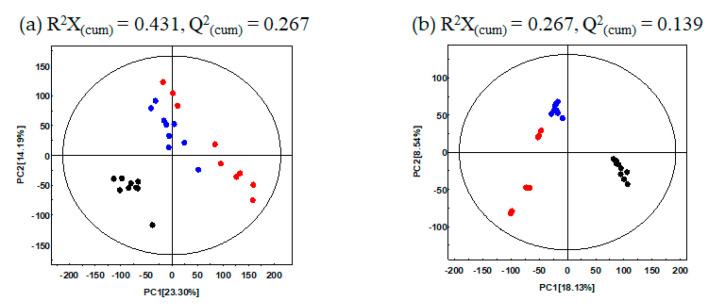
PCA score plots of Ginseng berry pomace (GBP) obtained after different ultra-high-pressure (UHP) processing treatments and analysis by (**a**) GC-TOF-MS; (**b**) UHPLC-LTQ-Orbitrap-MS/MS. ●: Non-UHP-treated GBP (CON), ●: UHP-treated GBP at 500 MPa (U500), ●: UHP-treated GBP at 600 MPa (U600).

In this study, to determine the metabolite variance associated with different UHP-treatments for GBP, comparative metabolite profiling was performed for the different pomace samples (CON, U500, U600) using GC-TOF-MS and UHPLC-LTQ-orbitrap-MS combined with multivariate analysis. The PCA score plot based on GC-TOF-MS and UHPLC-LTQ-orbitrap-MS datasets showed that the different types of UHP-treatments led to changes in metabolite distributions of GBP. Based on PCA analysis (Figure 1a), data for CON were found to be distinct from those of the UHP-treated pomace samples (U500 and U600) along PC1 (23.30%) and PC2 (14.19%). Similarly, the PCA plot derived from the UHPLC-LTQ-Orbitrap-MS/MS dataset showed that the metabolite profiles based on different UHP-treatments were separated by PC1 (18.13%) (Figure 1b). This stark separation within the datasets between the non-treated and treated pomace samples indicated their distinct metabolite profiles.

**Figure 2:**
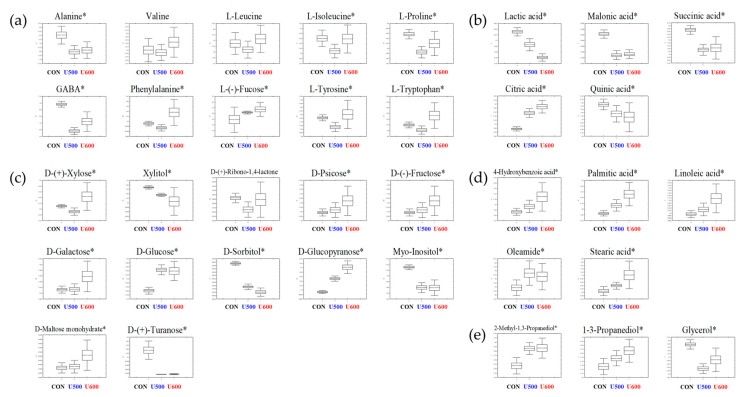
Box and whisker plots of significantly different metabolites among GBPs obtained after different UHP processing treatments. (**a**) Amino acids; (**b**) organic acids; (**c**) sugars and sugar alcohols; (**d**) fatty acids; and (**e**) others are shown. Relative contents of metabolites of GBP obtained after different processing treatments were calculated as a relative peak area in GC-TOF-MS analysis. The metabolite content corresponds to the relative peak area plotted in the *Y*-axis. (*Line*, mean; *box*, Standard error; *whisker*, standard deviation) The GBP obtained after different UHP processing treatments plotted in the *X*-axis. (CON, control; U500, 500 MPa; U600, 600 MPa) * *p*-value < 0.05.

To explore the primary metabolite profiles of the different pomace samples, all derivatized sample extracts were analyzed using GC-TOF-MS. To reveal metabolites that were discriminated according to the each of the UHP treatments, a PLS-DA model was applied (Figure S3a). The PLS-DA model of GC-TOF-MS analysis showed similar patterns to those obtained by the PCA score plot. The separation of each sample with R^2^X_(cum)_ = 0.462, R^2^Y_(cum)_ = 0.995, and Q^2^_(cum)_ = 0.945. Each sample was separated by PLS1 (22.13%) and PLS2(12.42%). The cross-validation results were R^2^Y intercept = 0.939, Q^2^Y intercept = 0.275, *p*-value = 2.0 × 10^−9^ (Figure S3b). A total of 35 significantly discriminant metabolites, including three alcohols, five organic acids, five fatty acids, ten amino acids, and twelve sugars and sugar alcohols, were selected for GBP based on the PLS-DA models with variable importance in projection values (VIP value > 0.7 and *p* < 0.05) derived from GC-TOF-MS (Table S3). Moreover, the relative abundance of the significantly discriminant metabolites under different UHP treatments was illustrated using box and whisker plots (Figure 2). Among them, 12 of the primary metabolites (alanine, L-proline, GABA, lactic acid, malonic acid, succinic acid, quinic acid, xylitol, D-sorbitol, *myo*-inositol, D-(+)-turanose, glycerol) exhibited a higher relative abundance in untreated pomace samples (CON). L-Isoleucine, phenylalanine, l-(−)-fucose, l-tyrosine, l-tryptophan, citric acid, d-(+)-xylose, d-psicose, d-(−)-fructose, d-galactose, d-glucose, d-glucopyranose, d-maltose monohydrate, 4-hydorxybenzoic acid, palmitic acid, linoleic acid, oleamide, stearic acid, 2-methyl-1,3-propanediol, and 1-3-propanediol were major components in both U500 and U600.

**Figure 3:**
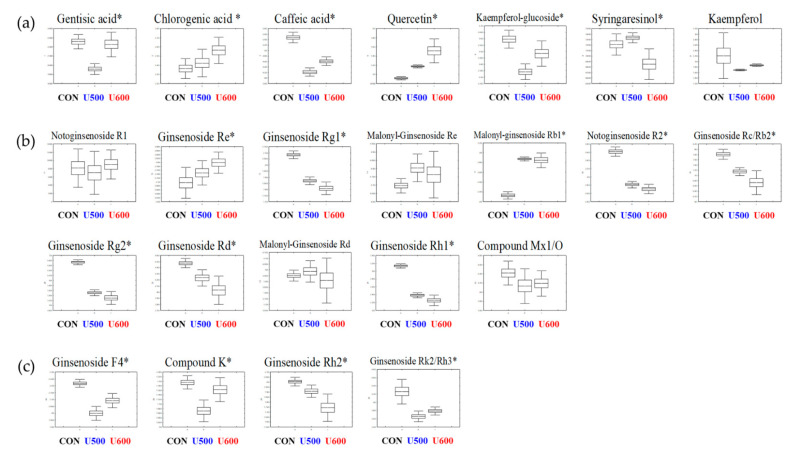
Box and whisker plots of significantly different metabolites among GBPs obtained after different UHP processing treatments. (**a**) Phenolic compounds; (**b**) polar ginsenosides; (**c**) less polar ginsenosides. Relative contents of metabolites of GBPs were calculated as a relative peak area in UHPLC-LTQ-Orbitrap-MS/MS analysis. The metabolite content corresponds to the relative peak area plotted in the *Y*-axis. (*Line*, mean; *box*, Standard error; *whisker*, standard deviation) The GBP obtained after different UHP processing treatments plotted in the *X*-axis. (CON, control; U500, 500 MPa; U600, 600 MPa) * *p*-value < 0.05.

To explore the secondary metabolites of the different pomace samples, all dried sample extracts were characterized by UHPLC-LTQ-Orbitrap-MS/MS. To reveal different secondary metabolites within GBP at different UHP treatments, both the VIP value above 0.7 and *p*-value below 0.05 of the variables were obtained from PLS-DA model (Figure S3c). The PLS-DA score plot showed similar pattern to the PCA score plot and the separation of each sample with R^2^X_(cum)_ = 0.309, R^2^Y_(cum)_ = 0.995, and Q^2^_(cum)_ = 0.918 in the GBP samples. Each pressure treatment was separated by PLS1 (18.09%) and PLS2 (8.16%). The results of cross-validation were represented with R^2^Y intercept = 0.942, Q^2^Y intercept = 0.106, *p*-value = 8.68× 10^−13^ (Figure S3d). A total of twenty-three significantly discriminant secondary metabolites, including seven phenolic compounds and sixteen ginsenosides, were selected for different GBP samples (Table S4). Among the pomace samples, most of the secondary metabolites had a higher relative abundance in CON, except chlorogenic acid, quercetin, ginsenoside Re, malonyl-ginsenoside Re, malonyl-ginsenoside Rb1, and malonyl-ginsenoside Rd (Figure 3).

### 2.2. Antioxidant Activity Assays of Different Pomace Samples

**Figure 4:**
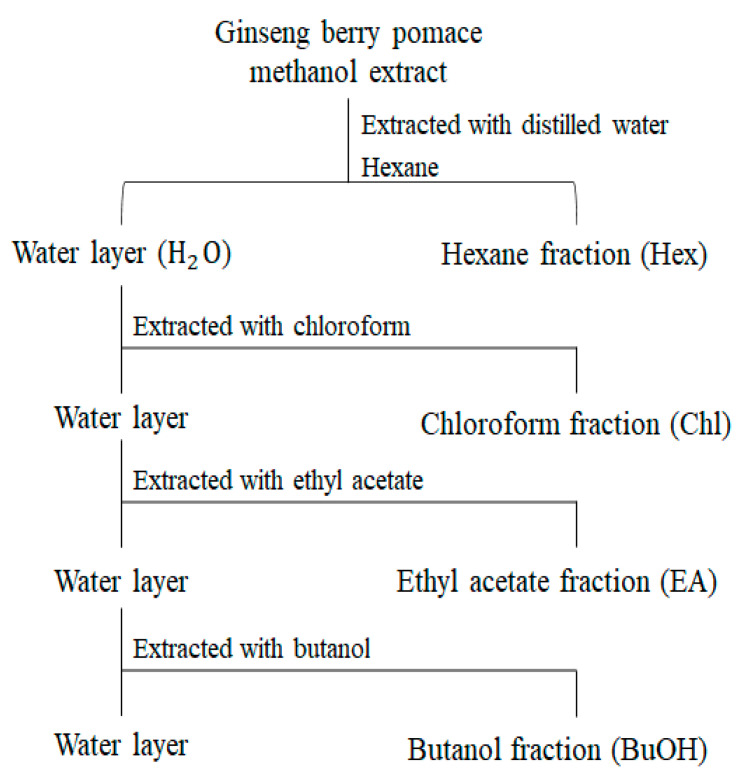
Flow chart of solvent–solvent extraction of GBP fractions with various solvents.

We evaluated the antioxidant activities (ABTS, DPPH, and FRAP) and TPC of the extracts representing the different UHP-processing treatments. As shown in Figure S4, there was no significant difference between the antioxidant activities of the extracts in the different UHP treatments. Furthermore, the TPC showed a similar tendency to that of antioxidative activities. Based on these results, we established three groups that were obtained after different UHP treatments using five solvents with different polarities (Figure 4) to determine the functional metabolites of pomace.

### 2.3. Relative Contents of Metabolites among the Solvent Fractions of GBPs

**Figure 5:**
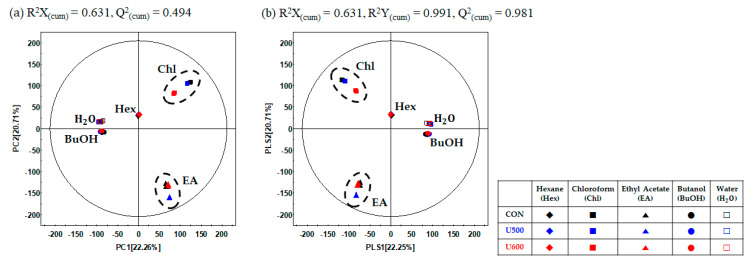
(**a**) PCA score plots; (**b**) PLS-DA score plots of different solvent fractions in GBP analyzed by UHPLC-LTQ-Orbitrap-MS/MS.

To explore the differences in the secondary metabolite profiles of the different solvent fractions, all dried sample extracts were characterized by UHPLC-LTQ-Orbitrap-MS/MS. Multivariate statistical analyses of the aligned datasets showed a distinct metabolomic pattern in the PCA (Figure 5a) and PLS-DA (Figure 5b) models. Based on PCA analysis, the patterns of the secondary metabolite profiles of the different solvent extracts were clustered into five groups (hexane, chloroform, ethyl acetate, butanol, and water) according to the polarity of PC1 (22.26%) and PC2 (20.71%) (Figure 5a). To reveal different secondary metabolites within GBP at different solvent fractions, both the VIP value above 0.7 and *p*-value below 0.05 of the variables obtained from PLS-DA model. Similar to the PCA results, the PLS-DA score plot (Figure 5b) could also be readily divided into five groups corresponding to the solvent polarity of the samples, along PLS1 (22.25%) and PLS2 (22.25%) and the separation of each samples with R^2^X_(cum)_ = 0.631, R^2^Y_(cum)_ = 0.991, and Q^2^_(cum)_ = 0.981 in the GBP samples. The results of cross-validation were represented with R^2^Y intercept = 0.228, Q^2^Y intercept = − 0.319, *p*-value = 2.90 × 10^−29^ (Figure S5).

**Figure 6:**
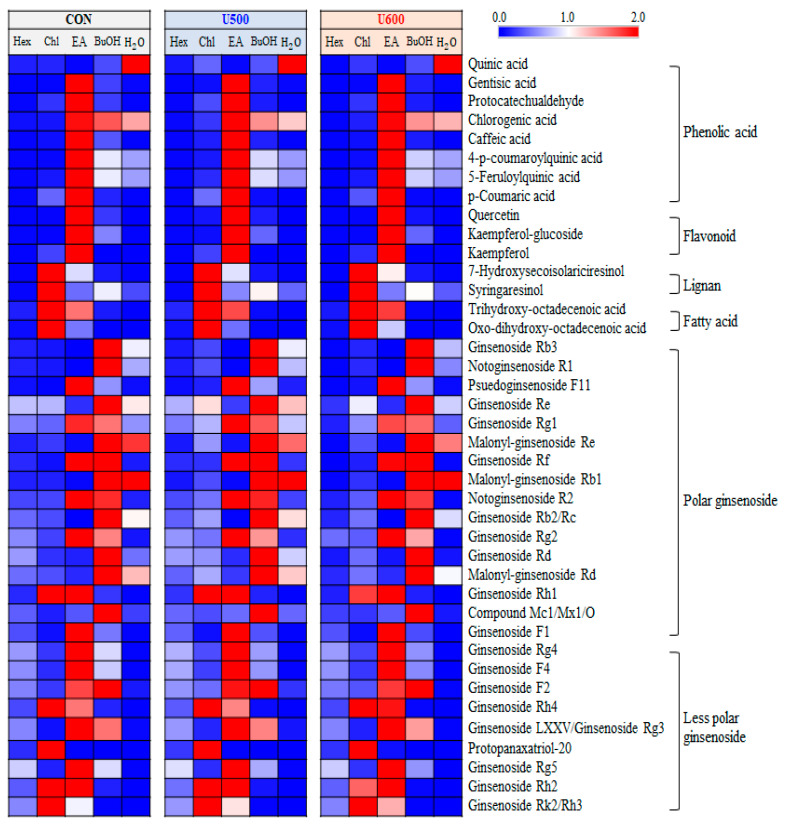
Heatmap analysis in five different solvent fractions of GBP samples derived from UHPLC-LTQ-Orbitrap-MS/MS data. The heatmap indicates the relative contents in secondary metabolites among different fractions. In this analysis, significantly discriminant metabolites were determined (VIP > 0.7 and *p* < 0.05).

A total of 40 significantly discriminant metabolites, including phenolic acids, flavonoids, lignans, fatty acids, and ginsenosides, were affected by solvent–solvent extraction (Table S5). Among the GBP samples, the differences with respect to solvent fractions in the relative content of significantly discriminant secondary metabolites were visualized using a heatmap (Figure 6). As shown in Figure 6, the GBP samples exhibited similarity in metabolite contents according to each sample, including CON, U500, and U600, but there was a difference in metabolites for each solvent fraction. Furthermore, seven phenolic acids and three flavonoids exhibited a higher relative abundance under the ethyl acetate fraction. The levels of two lignans and two fatty acids were higher in the chloroform fraction than in the other solvent fractions. Additionally, among the twenty-five ginsenosides, sixteen polar ginsenosides had a higher abundance under the ethyl acetate and butanol fractions, whereas the levels of nine less polar ginsenosides were higher in the chloroform and ethyl acetate fractions.

### 2.4. Analysis of Correlation between Metabolites in Solvent Fractions of GBP Extracts and Related Biochemical Phenotypes

**Figure 7:**
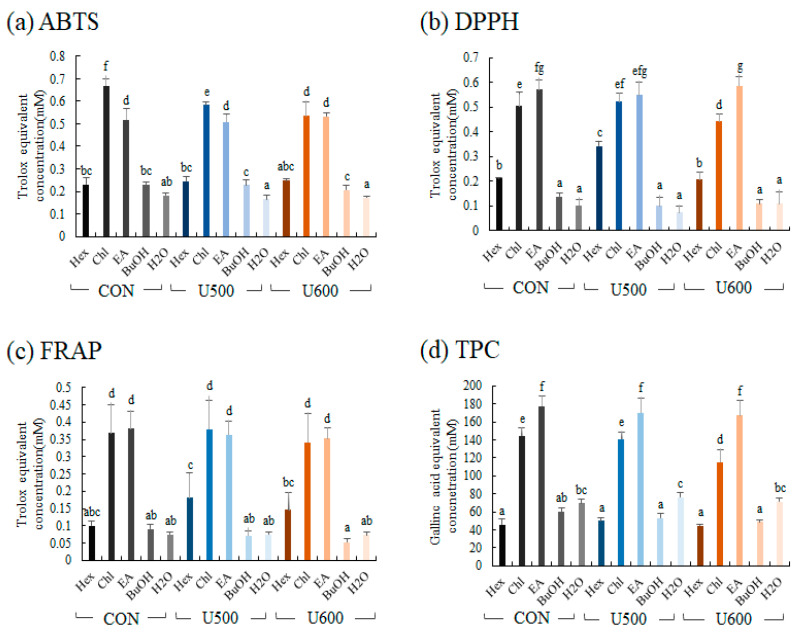
Antioxidant activity assay (**a**) ABTS; (**b**) FRAP; (**c**) DPPH; (**d**) total phenolic contents (TPC) of different solvent fractions. Values are expressed as the average of three biological replicates. The bar graph denoted by the same letter shows absence of statistical difference, according to Duncan’s multiple range test (*p* < 0.05). (Hex, Hexane fraction; Chl, Chloroform fraction; EA, Ethyl acetate fraction; BuOH, Butanol fraction; H_2_O, Water fraction).

To compare the bioactivities of the five solvent fractions, antioxidant activity (ABTS, DPPH, and FRAP) and TPC assays were performed. The antioxidant of five solvent fractions was represented as the standard of Trolox equivalent antioxidant activity (Figure 7). The differences among the groups obtained after the three different UHP treatments were not significantly different, and the antioxidant activities of chloroform and ethyl acetate were higher than those of the other fractions.

**Figure 8:**
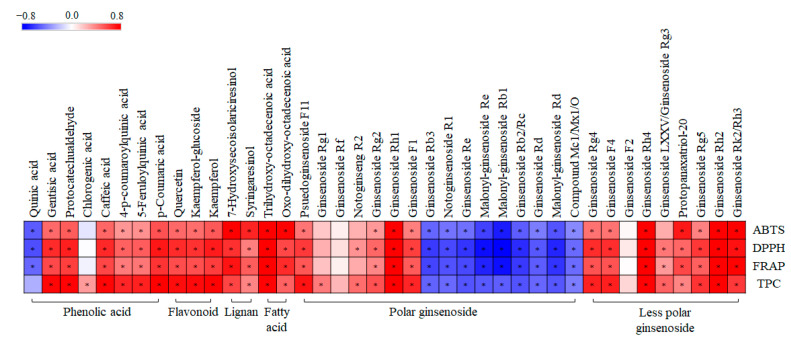
Correlation map between relative abundance of significantly discriminant metabolites and antioxidant assay (ABTS, DPPH, and FRAP), and total phenolic content (TPC) of different solvent fractions of GBP samples. Each square indicates Pearson’s correlation coefficient values (r). Red and blue represent positive (0 < *r* < 0.8) and negative (−0.8 < *r* < 0) correlations, respectively. *: *p*-value < 0.05.

Additionally, we performed a correlation analysis to evaluate the statistical relationship between 40 significantly discriminant metabolites and the observed tendencies of the related phenotypes, namely, ABTS, DPPH, FRAP, and TPC (Figure 8). Pearson’s correlation coefficients were calculated for the relative contents of the 40 significantly discriminant metabolites (Table S5) and their antioxidant activities. As shown in Figure 8, 30 metabolites exhibited positive correlations with the corresponding phenotypes, whereas the remaining 10 showed negative correlations. Interestingly, phenolic compounds (phenolic acid, flavonoid, lignan), fatty acids, less polar ginsenosides, and some polar ginsenosides showed significantly positive correlations with antioxidant activity. However, some polar ginsenosides showed significantly negative correlations with antioxidant activity.

## 3. Discussion

In this study, we demonstrated the relative contents of significantly different metabolites in GBP obtained after different UHP treatments. Additionally, we proved that the relative levels of metabolites in each solvent fraction of GBP were different and showed the correlation between significantly different metabolites and antioxidant activities. These findings suggest the potential of GBP for utilization in various industries.

Figure 2 and Figure 3 show the relative contents of metabolites in different GBPs, which are produced after UHP treatment and water extraction. The results revealed that UHP treatment altered the contents of some metabolites in GBPs [24,25]. Previously, Lee et al. [4] reported that the relative contents of the metabolites in GB were elevated by UHP treatment. Intriguingly, our results showed that CON contained higher levels of metabolites, especially phenolic compounds. After UHP treatment, prior to water extraction, levels of phenolic compounds were significantly improved compared with CON. One possible reason for this improvement is that UHP processing alters membrane permeability and disrupts cell walls, which, in turn, improves their extractability [26]. Furthermore, Zuorro et al. [27] reported that phenolic compounds were more soluble in organic solvents than deionized water. Therefore, when we extracted GBP using methanol, phenolic compounds that were less dissolved in water were extracted abundantly in the methanol extract.

Figure S4 shows the antioxidant activities of the different GBPs. The results revealed that the antioxidant activity of CON, which has high phenolic compound levels, did not show any significant difference compared to UHP-treated pomace samples. These results imply that the antioxidant activities of GBPs are influenced by various factors, including the level of phenolic compounds. Phenolic compounds are one of the most popular groups of phytochemicals, including phenolic acids, flavonoids, and lignans [28]. Previous studies have reported that the antioxidant activities of phenolic compounds are controlled by the quantity and position of their hydroxyl groups [29]. In addition to phenolics, ginsenosides, the major bioactive compounds of GB, also exhibit antioxidant activities [30]. Based on these findings, we suggest that the antioxidant activities of GBP are controlled by their phenolic compounds and ginsenosides. To determine the additional metabolites that affected the biological activities of GBP, we performed solvent–solvent extraction using various solvents, including hexane, chloroform, ethyl acetate, and butanol.

The solvent extraction method is commonly used for the extraction of metabolites from different medicinal plants and herbs. Both extraction yield and the biological activity of extracts are remarkably dependent on the type of solvent [31]. Therefore, a comparative study was performed for assessment of the ability of solvents to increase the utility of GBP as a biomaterial. For this purpose, solvent–solvent extraction was performed using various solvents (hexane, chloroform, ethyl acetate, and butanol) with different polarities. This process splits the higher quantities of bioactive compounds from the complex mixtures, which could be applied to separate the target compounds. This is an important step in bio-guided assays of new phytochemical ingredients [32]. Chua et al. [33] reported that highly polar substances, such as organic acids, polysaccharides, and proteins, might remain in the aqueous phase, while the other less polar compounds, including terpenoids, could be segregated into the organic phase. More specifically, the ethyl acetate fraction had the highest levels of saponins, which include ginsenosides. Additionally, few studies have reported that polyphenols and flavonoids, which are correlated with antioxidant and anti-inflammatory activities, are usually detected in ethyl acetate fractions [34]. Furthermore, Theodoridies et al. [35] reported that fatty acids and their esters were mostly observed in the chloroform fraction of grapes.

Interestingly, in GBP, we observed a positive correlation between antioxidant activity and ginsenosides, specifically less polar ginsenosides. Normally, ginsenosides are amphipathic, with four hydrophobic steroid-like ring structures and hydrophilic sugar moieties. The difference in the structure of ginsenosides is due to the position and type of sugar moieties. Furthermore, ginsenoside antioxidant activity is primarily affected by the type, linkage, and position of sugar moieties in ginsenosides [36]. Hydroxyl groups on sugar moieties can contribute to the scavenging activities of ginsenosides [37]. Based on polarity, ginsenosides are categorized into two groups, namely high- and low-polarity ginsenosides. Low-polarity ginsenosides are formed by the deglycosylation of major ginsenosides, such as ginsenoside Rb1, Rb2, Rc, Rd, and Re [38]. Since the sugar moieties in ginsenosides are responsible for their antioxidant activity, deglycosylation, which transforms major ginsenosides into low-polarity ginsenosides, leads to the elevation of ginsenoside antioxidant activity. Previously, several studies have reported that less polar ginsenosides have better bioactivity than the major ginsenosides [39]. In agreement with the findings illustrated in Figure 8, Yao et al. [40] have stated that less polar ginsenosides demonstrate a significant positive correlation with antioxidant activity.

In this study, both ethyl acetate and chloroform fractions were useful for the extraction of functional metabolites that could influence antioxidant activity. Furthermore, due to its low boiling point and toxicity, the ethyl acetate fraction can safely be used in the food and cosmetic industry for the extraction of metabolites. Moreover, ethyl acetate is inexpensive, which is good from an economical viewpoint [41]. Chloroform is non-inflammable and is used for extraction of the active constituents from shrubs and other related plant species [42].

In this study, we provide the in vitro antioxidant activity and a correlation with the metabolites of GBP. However, the in vitro antioxidant capacity of GBP is only an approximate reflection of their in vivo effect due to the differences in bioavailability within the digestive tract and the metabolism of compounds [43]. Further, a limited number of studies demonstrated the in vivo antioxidant activity of metabolites extracted from ginseng berry. Martins et al. [44] analyzed the in vivo antioxidant activity of phenolic compounds (*p*-coumaric acid, gentisic acid, protocatechuic acid, caffeic acid, kaempferol) and these results proved that the ginseng berry metabolites have a positive correlation with different antioxidant activities. Additional, syringaresinol, which is only found in GBs [7], was reported the in vivo antioxidant activity in agreement with our findings [45]. Further, some studies reported that the in vivo antioxidant activity of ginsenoside. As shown in Figure 8, ginsenosides Rg1, which are positively correlated with bioactivity, protect cardiomyocyte from hypoxia/reoxygenation oxidative injury [46]. Ginsenoside Rh2 also has a positive correlation with bioactivity (Figure 8). Zeng et al. [47] reported that the ginsenoside Rh2 has an antioxidant effect in vivo and restores the balance of the antioxidant defense system by suppressing oxidative stress. Through this, we prove that some metabolites still have an antioxidant activity in vivo. This research supports the potential of GBP as a beneficial biomaterial for human.

## 4. Materials and Methods

### 4.1. Chemicals and Reagents

HPLC-grade solvents, including water, methanol, hexane, chloroform, ethyl acetate, and butanol, were purchased from Fisher Scientific (Pittsburgh, PA, USA). All standard compounds and analytical grade reagents used in this study were obtained from Sigma Chemical Co. (St. Louis, MO, USA).

### 4.2. Materials

Raw samples of GB belong to the Araliaceae family and consist of the berry skin and pulp without seeds. GBP was obtained as a by-product after the water extraction process of the GB raw material. Prior to water extraction, dispersion was subjected to UHP treatment at different pressure levels using pilot-scale HHP (BaoTou Kefa Co., Ltd., BaoTou, China). Following UHP treatment, the dispersion was mixed with distilled water, filtered, and the precipitate was collected. The resultant precipitate (pomace) was freeze-dried, powdered using a mortar and pestle, and stored at −20 °C for further analysis. The UHP-untreated pomace sample was obtained only after the water extraction process and was used as a control (CON). According to the pressure levels, the GBP samples were labeled U500 and U600, respectively. U500 was the first sample that was subjected to two repeated treatments of UHP at 500 MPa/5 L, and subsequently, the applied pressure was increased at a rate of 2 MPa/s to 500 MPa, and the decompression time was approximately 10 s. Tap water was used as a pressure transmission fluid. The second sample, U600, was obtained after using a single UHP treatment at 600 MPa/5 L.

### 4.3. Sample Preparation

Each sample (100 mg) was extracted with methanol (1 mL) using the MM400 mixer mill (Retsch GmbH, Haan, Germany) at a frequency of 30 s^−1^ for 10 min, followed by sonication for 5 min (Hettich Zentrifugen Universal 320, Tuttlingen, Germany). After sonication, the sample dispersion was centrifuged at 17,000 rpm for 10 min at 4 °C, and the resultant supernatants were filtered through a 0.2-μm PTFE filter (Chromdisc, Daegu, Korea). Then, the soluble filtrates were dried using a speed-vacuum concentrator (Biotron, Seoul, Korea) and stored at −20 °C for further analysis. The samples were analyzed using three biological replicates for each sample. The quality control (QC) samples were made by using the pooled mixture from 10 μL of each sample (biological replicate). The analytical samples were analyzed in blocks of 10 runs for followed by an intermittent QC analysis to ensure the data quality and robustness of the method.

### 4.4. GC-TOF-MS Analysis

Prior to GC-TOF-MS analysis, each GBP sample extract was subjected to two derivatization reactions, following a method described by Lee et al. [4]. First, oximation was performed by adding 50 μL of methoxyamine hydrochloride in pyridine (20 mg/mL) to each dried sample and by incubating the reaction at 30 °C for 90 min. Next, silylation was performed by adding 50 μL of N-methyl-N-(trimethylsilyl) trifluoroacetamide and by incubating the reaction at 37 °C for 30 min. The derivatized samples were filtered using the Milex GP 0.22-μm filter before analysis, and the final concentration of the derivatized sample was 20 mg/mL.

GC-TOF-MS analysis was performed using the Agilent 7890A GC system (Agilent Technologies, Palo Alto, CA, USA) equipped with the Agilent 7693 autosampler and Pegasus high-throughput (HT)_TOF-MS program (Leco Corp., St. Joseph, MI, USA). The metabolites were separated using an Rtx-5MS column (30 m × 0.25 mm; 0.25 µm; Restek Corp. Bellefonte, PA, USA) and the operational parameters were adapted from a study reported by Lee et al. [48]. The chromatograms of samples are shown in Figure S1.

### 4.5. UHPLC-LTQ-Orbitrap-MS/MS Analysis

For UHPLC-LTQ-Orbitrap-MS/MS analysis, each dried sample (10 mg/mL) was dissolved in 100% methanol and used. UHPLC-LTQ-Orbitrap-MS/MS analysis was performed using a UHPLC system equipped with the Vanquish binary pump H system (Thermo Fisher Scientific, Waltham, MA, USA) coupled with an auto-sampler and column compartment. The chromatographic separation was performed on the Phenomenex KINETEX^®^ C18 Column (100 × 2.1 mm, 1.7 μm; Torrance, CA, USA) and the operational parameters were adapted from a study reported by Lee et al. [48]. The chromatograms of samples are shown in Figure S2.

### 4.6. Solvent–Solvent Extraction

The solvent–solvent extraction process was performed following the method described by Lim et al. [49] with minor modifications. Initially, the dried methanolic extract (200 mg) of pomace was redissolved in 8 mL of deionized water. For the aqueous extract, 16 mL of organic solvents, including hexane, chloroform, ethyl acetate, butanol, and deionized water, were added serially (Figure 4). After the addition of organic solvents, dispersion was filtered, and the supernatant in different solvents was recovered. Finally, the supernatant in different solvents was evaporated using a speed-vacuum concentrator (Biotron, Seoul, Korea). The extract yield of the solvent fractions is shown in Table S1. After this process, we used the same LC-MS method for analysis as described above (2.5 mg/mL) and compared the significantly different metabolites with solvent fractions.

### 4.7. Data Processing and Multivariate Statistical Analysis

The raw data files from GC-TOF-MS and UHPLC-LTQ-Orbitrap-MS/MS were converted into a computable document form (.cdf) format using the LECO Chroma TOF software v.4.44 (Leco Co., USA) and Thermo Xcalibur v.2.2 (Thermo Fisher Scientific, San Jose, CA, USA), respectively. After conversion, the software MetAlign (http://www.metalign.nl) was used to preprocess the netCDF data to obtain baseline correction, peak alignment, peak detection, accurate masses, and normalized peak intensities [50]. The parameters of MetAlign were set according to the specific scaling requirements and chromatographic and mass spectrometric conditions used in the experiments (Table S2). Subsequent data, which contained the sample name and peak area information as variables, were transferred to an Excel spreadsheet, and multivariate statistical analyses were executed using the SIMCA-P+ 12.0 software (Umetrics, Umea, Sweden). Furthermore, both unsupervised principal component analysis (PCA) and supervised partial least-square discriminant analysis (PLS-DA) were performed to compare the different metabolites of the samples. To decrease the sample size and improve data interpretability by selecting influential variables, PLS-DA was used. Based on this, the significant discriminant metabolites were selected uniformly at VIP > 0.7 and *p*-value < 0.05. In PLS-DA model, the R^2^X and R^2^Y represent a fraction of the variance of the X and Y matrix variables explained by the model. Q^2^ represented the predictive capacity of the model. R^2^X_(cum)_ and R^2^Y_(cum)_ is the cumulative fraction of the sum of squares of X and Y, explained including the selected component. Q^2^_(cum)_ represent cumulative predicted variation in the Y matrix. Cross-validation analysis of PLS-DA results derived from GC-TOF-MS and UHPLC-LTQ-Orbitrap-MS/MS analyses were summarized in Figure S3 and S5 [51]. This analysis indicates the prediction accuracy, fitness, and the quality of the model. The values of R^2^Y and Q^2^ close to 1.0 were indicative of a valid model with a high robustness. The selected metabolites were identified by comparing their retention times and mass fragment patterns with standard compounds, in-house library data, references, and various databases, such as the National Institutes of Standard and Technology (NIST) Library (v.2.0, 2011, FairCom, Gaithersburg, MD, USA), the Dictionary of Natural Products (v.16:2, 2007, Chapman and Hall, USA), Wiley 8, and the Human Metabolome Database (HMDB; http://www.hmdb.ca/).

Box-whisker plots were performed using the relative peak area of metabolites by STATISTICA (version 7.0, StatSoft Inc., Tulsa, OK, USA). For the bioactivity assays, differences were tested by analysis of variance and Duncan’s multiple range tests using PASW Statistics 18 (SPSS Inc, Chicago, IL, USA). Correlations between metabolites and bioactivity assays were calculated by Pearson’s correlation coefficient test using PASW Statistics 18. The *p*-values for different metabolite-based clusters were determined by one-way ANOVA using STATISTICA.

### 4.8. Antioxidant Activity Analysis

ABTS (2,2′-azino-bis (3-ethylbenzothiazoline-6-sulfonic acid), ferric reducing antioxidant power (FRAP), and DPPH (2,2-diphenyl-1-picryl-hydrazyl) radical scavenging assays were performed to measure the in vitro antioxidant activities of the different GBP samples (2.5 mg mL^−1^ methanol), following the procedure reported by Lee et al. [4].

### 4.9. Total Phenolic Content Assay

The total phenolic content (TPC) assay of different GBP samples was performed in two steps. First, 20 μL of the GBP sample was added to 100 μL of 0.2 N Folin–Ciocalteu’s phenol reagent, followed by incubation for 5 min at room temperature in the dark. Next, 80 μL of 7.5% Na_2_CO_3_ was added to the samples, and the resulting reaction mixtures were incubated for 60 min. Finally, the absorbance of the samples was measured at 750 nm using a microplate reader (Spectronic Genesys 6). The assay results were expressed in terms of the gallic acid equivalent of the activity (μg mL^−1^), and as the mean value of three analytical replicates.

## 5. Conclusions

In the present study, we used metabolomic approaches to compare the remaining metabolites in GBP after UHP treatment. Additionally, we performed solvent–solvent extractions to determine the antioxidant activities and diverse metabolites of GBP using further fractionation by various solvents. From these results, we provide the potential of GBP as a beneficial antioxidant. Instead of ginseng berry, pomace can replace it in cosmetic or food industries, and then it is good from an economical viewpoint compared with GB. Additionally, these results demonstrate that UHP treatment, followed by solvent extraction, is useful for the isolation of desired metabolites and advantageous for industrial applications. Through this, we believe that GBP is not a “useless by-product” anymore but can be used as a beneficial biomaterial. If the industry re-uses it as a functional ingredient, they can also reduce the environmental issues which are caused by discarded by-product. However, a limitation of the present study is that the quantification of functional metabolites in GBP has not been performed to date. Therefore, it is necessary to evaluate the contents of functional components for further applications.

## Data Availability

The data presented in this study are available on request from the corresponding author.

## Supplementary Materials

The following are available online, Table S1: The yield of fractions obtained using various solvents for GBP. Figure S1: The GC-TOF-MS chromatogram of GBP obtained after different UHP processing treatments (a) CON, control; (b) U500, 500 MPa; (c) U600, 600 MPa is shown. Figure S2: The UHPLC-LTQ-Orbitrap-MS/MS chromatogram of GBP obtained after different UHP processing treatments (a) CON, control; (b) U500, 500 MPa; (c) U600, 600 MPa is shown. Table S2: MetAlign settings used to automatically process the experimental dataset. Figure S3: PLS-DA score plots and validation plots for GBP obtained after different UHP processing treatments. (a) PLS-DA score plots (GC-TOF-MS); (b) validation plot (GC-TOF-MS); (c) PLS-DA score plot (UHPLC-LTQ-Orbitrap-MS/MS); (d) validation plot (UHPLC-LTQ-Orbitrap-MS/MS). Table S3: Differential metabolites in the GBP obtained after different UHP processing treatments and GC-TOF MS analyses. Table S4: Differential metabolites in the GBP obtained after different UHP processing treatments and UHPLC-LTQ-Orbitrap-MS/MS analyses. Figure S4: Antioxidant activity assay (a) ABTS; (b) FRAP; (c) DPPH; (d) total phenolic content (TPC) of GBP obtained after different UHP processing treatments. Figure S5: Validation plots of different solvent fractions of GBP analyzed by UHPLC-LTQ-Orbitrap-MS/MS. Table S5: Differential metabolites in different solvent fractions of GBP obtained after UHPLC-LTQ-Orbitrap-MS/MS analyses.

## Sample Availability

Samples of the compounds are not available from the authors.
