# Supplementary material for: Evaluation of Metabolite Profiles of Ginseng Berry Pomace Obtained after Different Pressure Treatments and Their Correlation with the Antioxidant Activity
Source: Molecules. 2021 Jan 8;26(2):284. doi: 10.3390/molecules26020284 (PMC7827211; doi:10.3390/molecules26020284)
Supplement: Supplementary file 1 [file molecules-26-00284-s001.pdf]

Supplementary Information

Table S1. The yield of fractions obtained using various solvents for GBP

Fraction	Yield (%)
Hexane	15.58
Chloroform	5.74
Ethyl acetate	4.70
Butanol	50.00
Water	46.14

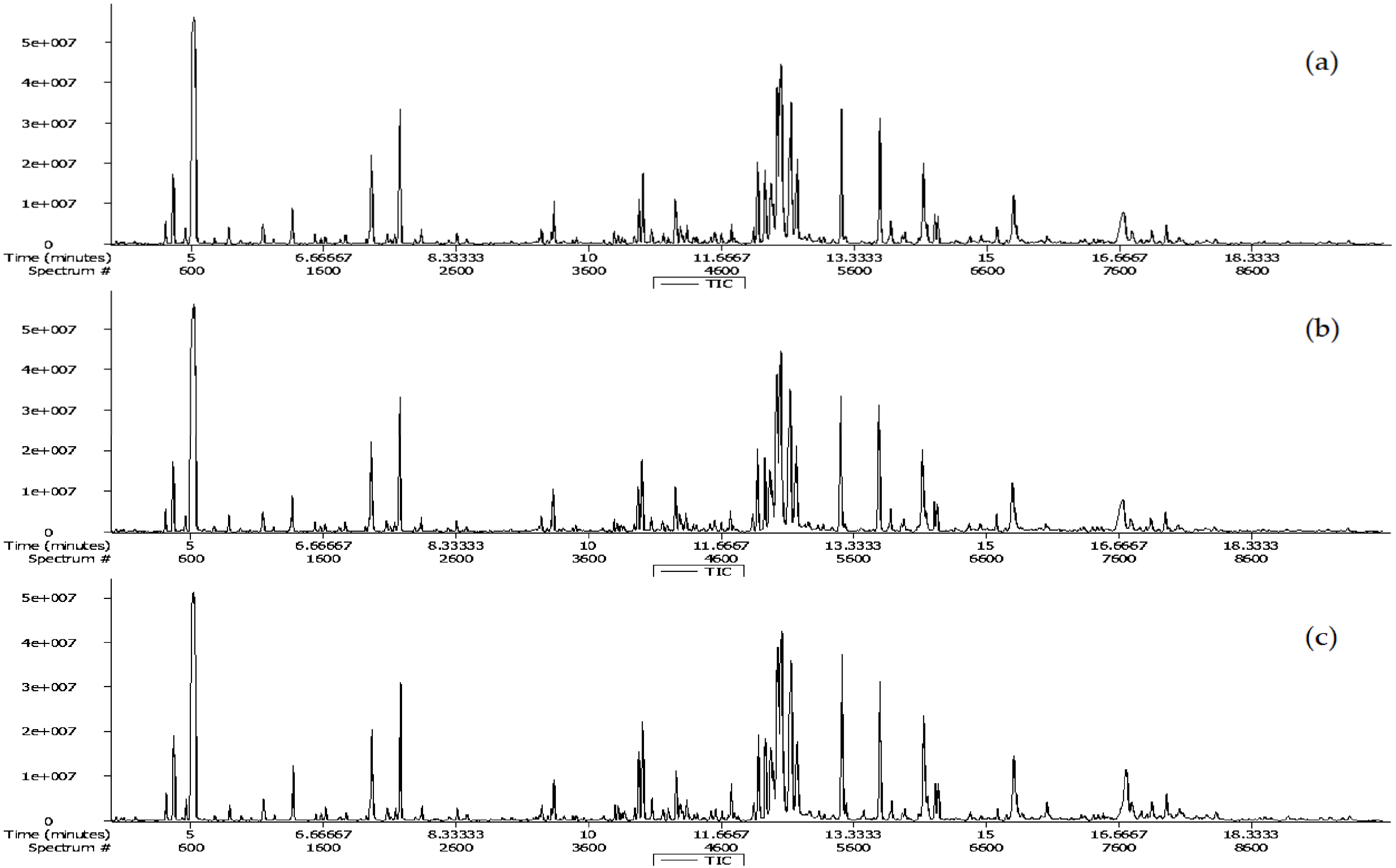

Figure S1. The GC-TOF-MS chromatogram of GBP obtained after different UHP processing treatments (a) CON, control; (b) U500, 500MPa; (c) U600, 600MPa is shown.

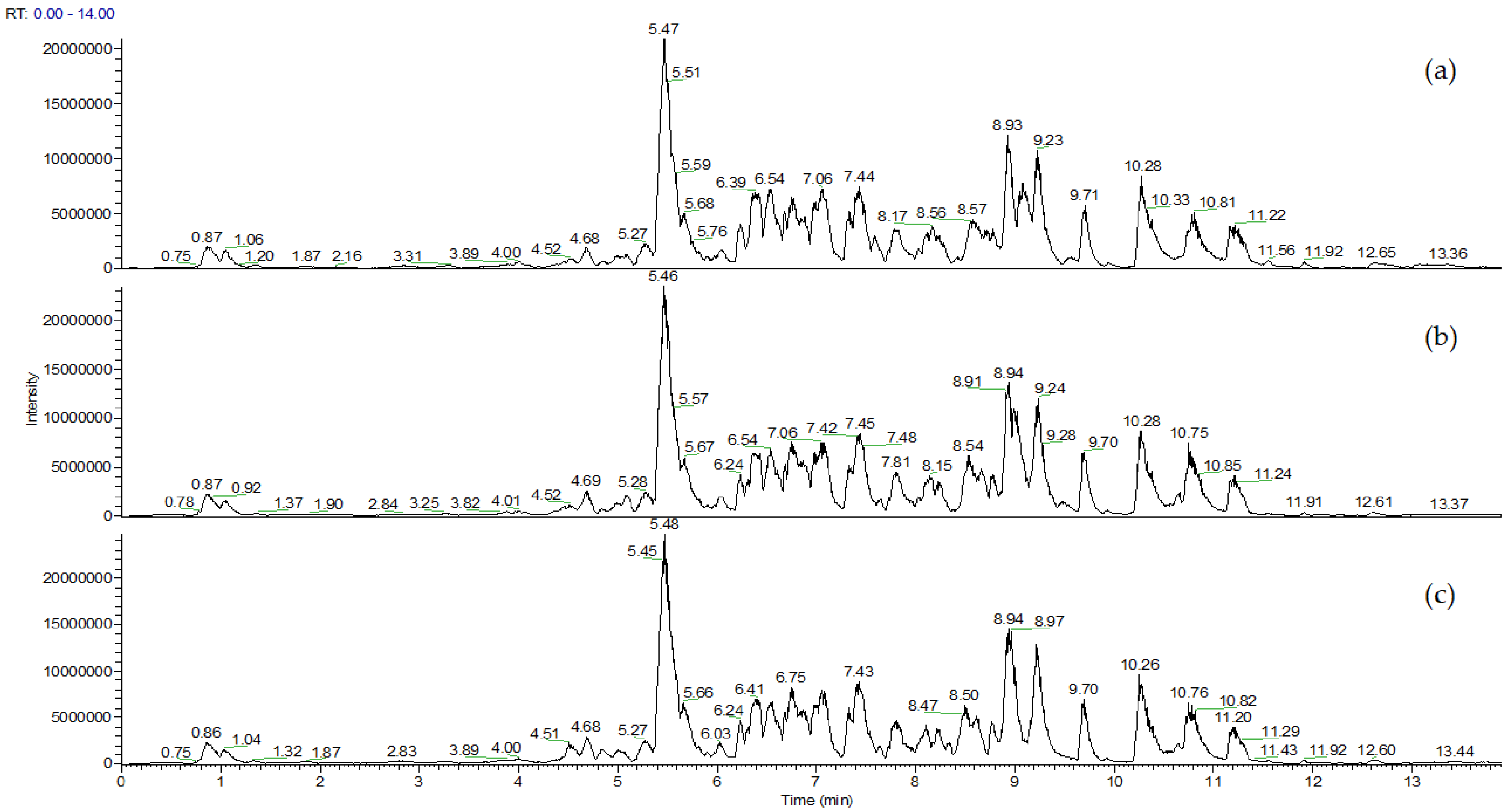

Figure S2. The UHPLC-LTQ-Orbitrap-MS/MS chromatogram of GBP obtained after different UHP processing treatments (a) CON, control; (b) U500, 500MPa; (c) U600, 600MPa is shown.

Table S2. MetAlign settings used to automatically process the experimental dataset.

Parameter	Value	
	GC-TOF-MS	UHPLC-LTQ-Orbitrap-MS/MS
Retention begin (scan nr.)	1	1
Retention end (scan nr.)	9,600	9,600
Maximum amplitude	10,000,000	10,000,000
Peak slope factor (x Noise)	1	1
Peak threshold factor (x Noise)	2	2
Peak threshold (Abs. Value)	30	20
Average peak width at half height (Scans)	80	30
Scaling Options	None	None
Maximum shift per scan	50	30
Select min nr per peak set	9	9

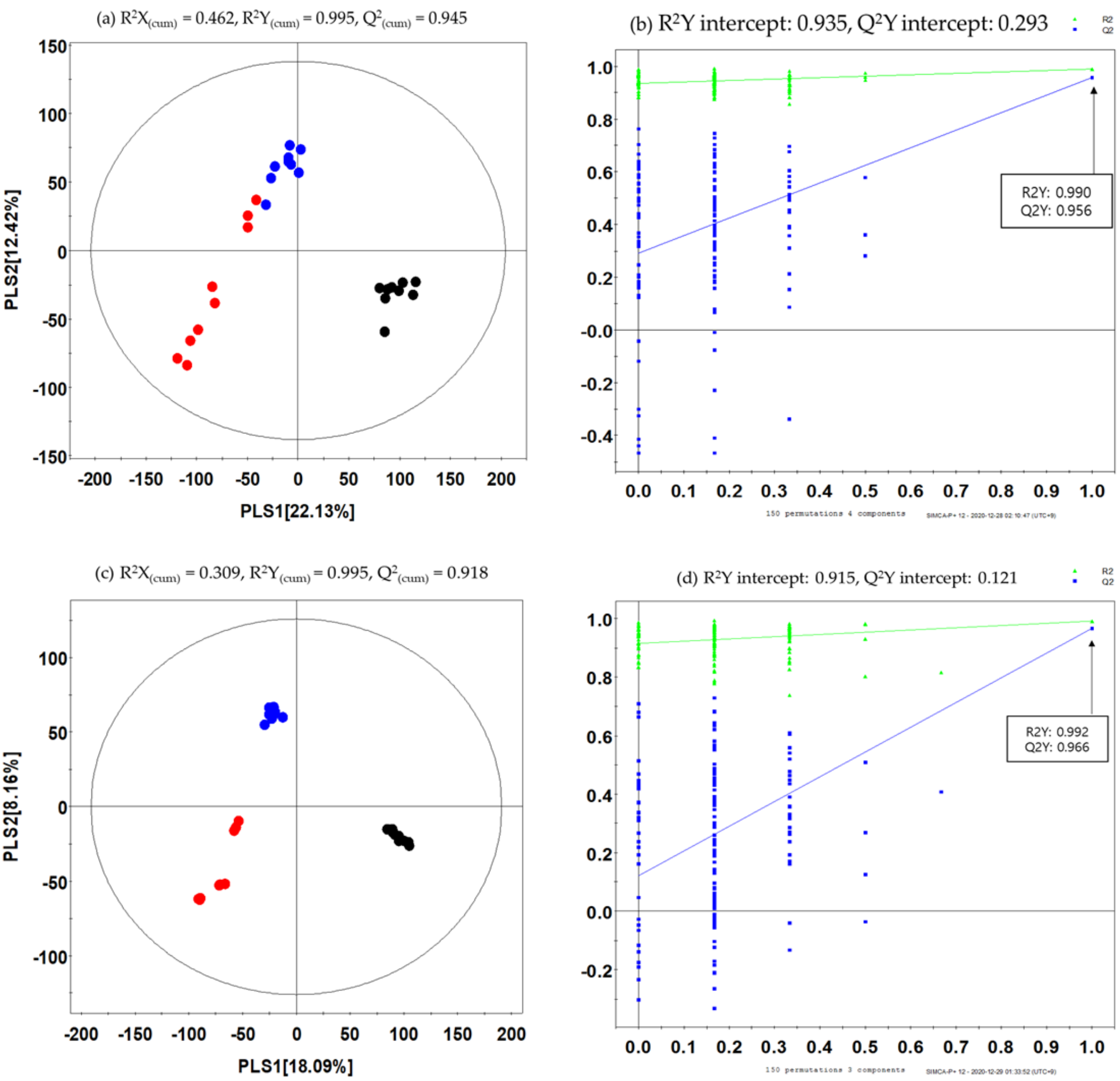

Figure S3. PLS-DA score plots and validation plots for GBP obtained after different UHP processing treatments. (a) PLS-DA score plots (GC-TOF-MS); (b) validation plot (GC-TOF-MS); (c) PLS-DA score plot (UHPLC-LTQ-Orbitrap-MS/MS); (d) validation plot (UHPLC-LTQ-Orbitrap-MS/MS). ●: Non-UHP-treated GBP (CON), ●: UHP-treated GBP at 500 MPa (U500), ●: UHP-treated GBP at 600 MPa (U600). The Y axis of validation plot represent R^2Y (green colored triangle) and Q^2Y (blue colored squared) for every model, and the X axis indicates the correlation coefficient between original and permuted response data. The Y intercepts of plot for the R^2Y and Q^2Y in each model were represented in number.

Table S3. Differential metabolites in the GBP obtained after different UHP processing treatments and GC-TOF-MS analyses

RT (min)	Metabolites[a]	Mass	MS fragments[b]	TMS	Ref.[c]	VIP1	VIP2
			Amino acids				
5.46	Alanine	116	73, 86, 100, 116, 133, 147, 190	2	Lib/STD	1.4	1.4
6.68	Valine	144	73, 86, 100, 133, 144, 218	2	Lib/STD	0.5	0.8
7.25	L-Leucine	158	73, 102, 116, 147, 158, 218, 232	2	Lib/STD	0.3	1
7.46	L-Isoleucine	158	73, 100, 114, 147, 158, 218, 232	2	Lib/STD	0.2	1.3
9.53	L-Proline	156	73, 100, 133, 147, 156, 230, 258	2	Lib/STD	0.9	1.5
9.55	GABA	174	73, 86, 147, 174, 216, 246, 304	3	Lib/STD	1.4	1.9
10.36	Phenylalanine	218	73, 100, 147, 192, 218, 266	2	Lib/STD	0.9	1.5
11.11	L-(-)-Fucose	160	73, 103, 117, 147, 160, 277	4	Lib/STD	1.1	0.8
12.6	L-Tyrosine	218	73, 100, 179, 218, 280, 354	3	Lib/MS	0.2	1.4
14.38	L-Tryptophan	202	73, 100, 130, 147, 202, 218, 291	3	Lib/STD	0.7	1.5
			Organic acid				
5.04	Lactic acid	219	73, 101, 117, 133, 147, 191, 219	2	Lib/STD	2	1.6
6.55	Malonic acid	147	73, 99, 117, 133, 147, 233	2	Lib/STD	1.9	1.7
7.62	Succinic acid	247	73, 86, 129, 147, 172, 218, 247	2	Lib/STD	1.5	1.5
11.78	Citric acid	273	73, 133, 147, 183, 211, 257, 273	4	Lib/STD	2	1.5
12.12	Quinic acid	255	73, 133, 147, 239, 255, 283	5	Lib/STD	1.1	0.8
			Sugar and sugar alcohol				
10.67	D-(+)-Xylose	217	73, 103, 147, 189, 205, 217, 307	4	Lib/STD	0.7	1.4
10.93	Xylitol	217	73, 103, 129, 147, 157, 189, 217	5	Lib/STD	1.3	1
11.23	D-(+)-Ribono-1,4-lactone	246	73, 117, 133, 147, 204, 217, 259	3	Lib/MS	0.2	1.1
12.21	D-Psicose	217	73, 103, 147, 189, 217, 277, 307	5	Lib/MS	1	0.9
12.22	D-(-)-Fructose	307	73, 103, 147, 189, 217, 307, 364	5	Lib/STD	1	0.9
12.37	D-Galactose	409	73, 160, 205, 229, 319	5	Lib/STD	1	1.1
12.55	D-Glucose	160	73, 103, 129, 147, 160, 205, 319	5	Lib/STD	1.7	1.5
12.62	D-Sorbitol	319	73, 103, 117, 147, 205, 217, 319	6	Lib/STD	2.1	1.7
13.23	D-Glucopyranose	204	73, 103, 129, 147, 204, 217	5	Lib/STD	2.1	1.6
13.65	Myo-Inositol	217	73, 129, 147, 191, 217, 265, 318	6	Lib/STD	1.8	1.6
17.07	D-Maltose monohydrate	361	73, 149, 169, 204, 217, 243, 271	8	Lib/STD	1	1
17.25	D-(+)-Turanose	361	73, 103, 147, 169, 191, 217, 319	8	Lib/MS	1.9	1.7
			Fatty acids				
10.31	4-Hydroxybenzoic acid	223	73, 126, 179, 193, 223, 267, 282	2	Lib/STD	1.2	1
13.17	Palmitic acid	313	73, 117, 145, 159, 201, 269, 313	1	Lib/STD	1.6	1.3
14.2	Linoleic acid	337	75, 95, 109, 129, 150, 262, 337	1	Lib/STD	1.2	1.1
14.35	Stearic acid	132	75, 117, 129, 145, 185, 201, 341	1	Lib/STD	1.3	1.1
15.34	Oleamide	144	75, 91, 116, 131, 198, 226, 338	1	Lib/STD	0.9	1
			Others				
4.78	2-Methyl-1,3-Propanediol	117	73, 87, 101, 117, 133, 147, 219	2	Lib/MS	1.5	1.3
4.92	1-3-Propanediol	130	73, 103, 115, 130, 147, 177, 205	2	Lib/MS	1.3	1
7.26	Glycerol	205	73, 103, 117, 133, 147, 205, 218	3	Lib/STD	1.3	1.7

[a] Metabolites identified based on variable importance projection (VIP) analysis results (with a cut-off value of 0.7) and $p < 0.05$. [b] MS fragmentation is the fragmentation of the tentative compound. [c] The MS spectrum was consistent with those of NIST and in-house libraries. The standard compound (STD) mass spectrum was similar to that of the standard compounds. Retention time, RT; trimethylsilyl, TMS.

Table S4. Differential metabolites in the GBP obtained after different UHP processing treatments and UHPLC-LTQ-Orbitrap-MS analyses

Tentative metabolites[a]	RT (min)[b]	$[M-H]^-$	$[M+H]^+$	M.W.[c]	Molecular Formula	Delta ppm	MS/MS Fragments	Ref.[d]
Gentisic acid	1.86	153.0565	155.0703	154	C8H10O3	4.9	153>134, 109	[1]
Chlorogenic acid	3.83	353.089	355.1021	354	C16H18O9	3.3	191>179>135	[2]
Caffeic acid	4.01	179.0355	181.0494	180	C9H8O4	4.3	179>135	[3]
Quercetin	4.93	301.0364	303.05	302	C15H10O7	3.4	301>273>245	[4]
Kaempferol-glucoside	5.08	447.0945	449.1072	448	C21H20O11	2.8	447>284>255	[4]
Syringaresinol	5.11	417.1574	431.1122[f]	418	C22H26O8	4.7	417>371>327>167	webDB[e]
Notoginsenoside R1	5.28	977.5337[g]	955.5242[f]	932	C47H80O18	3.5	977>931>799,637,475	[5]
Ginsenoside Re	5.48	945.5388	969.5378[f]	946	C48H82O18	-4.3	945>637>475	[5]
Ginsenoside Rg1	5.49	845.4898[g]	801.4979	800	C42H72O14	-0.7	845>799>637>475>391	[5]
Malonyl-Ginsenoside Re	5.67	1031.5425	1055.5389[f]	1032	C51H84O21	-1.4	1031>987>945, 927	[6]
Malonyl-ginsenoside Rb1	6.31	1193.5944	1195.6102	1194	C57H94O26	-1.4	1193>1149>1107	[7]
Notoginsenoside R2	6.4	769.476	793.4691[f]	770	C41H70O13	2.1	815>769>637>475	[7]
Ginsenoside Rc/Rb2	6.45	1077.584	1101.5801[f]	1078	C53H90O22	1.1	1077>945>783, 621	[5]
Ginsenoside Rg2	6.54	829.4957[g]	807.4864[f]	784	C42H72O13	0.3	829>783>647, 475	[5]
Kaempferol	6.55	285.041	287.0544	286	C15H10O6	2.1	285>257, 150>107	[4]
Ginsenoside Rd	6.69	991.5488[g]	1015.548[f]	946	C48H82O18	0.5	991>945>783, 621	[5]
Malonyl-Ginsenoside Rd	6.77	1031.5422	1033.5605	1032	C51H84O21	-1	1031>987>945	[5]
Ginsenoside Rh1	6.8	683.4388[g]	661.4272[f]	638	C36H62O9	1.8	683>637>475	[5]
Compound Mx1/O	7.06	961.5392[g]	917.5463	916	C47H80O17	1.6	961>915>753, 621	STD[e]
Ginsenoside F4	7.4	765.4784	767.4924	766	C42H70O12	-1.4	765>617>457	[7,8]
Compound K	8.67	667.4442[g]	645.4318[f]	622	C36H62O8	2.3	667>621>459	[7]
Ginsenoside Rh2	8.85	667.4442[g]	645.4347[f]	622	C36H62O8	-0.6	667>621, 369	[7]
Ginsenoside Rk2/Rh3	9.79	649.4328[g]	627.421[f]	604	C36H60O7	1.1	649>603>585, 445	[8]

[a] The different metabolites were identified from the variable important in projection (VIP) analysis with a cut-off value of 0.7 and *p*-value < 0.05. [b] Retention time. [c] Molecular weight. [d] References. [e] Standard of ginsenoside. [f] $[M+Na]^+$. [g] $[M+FA-H]^-$

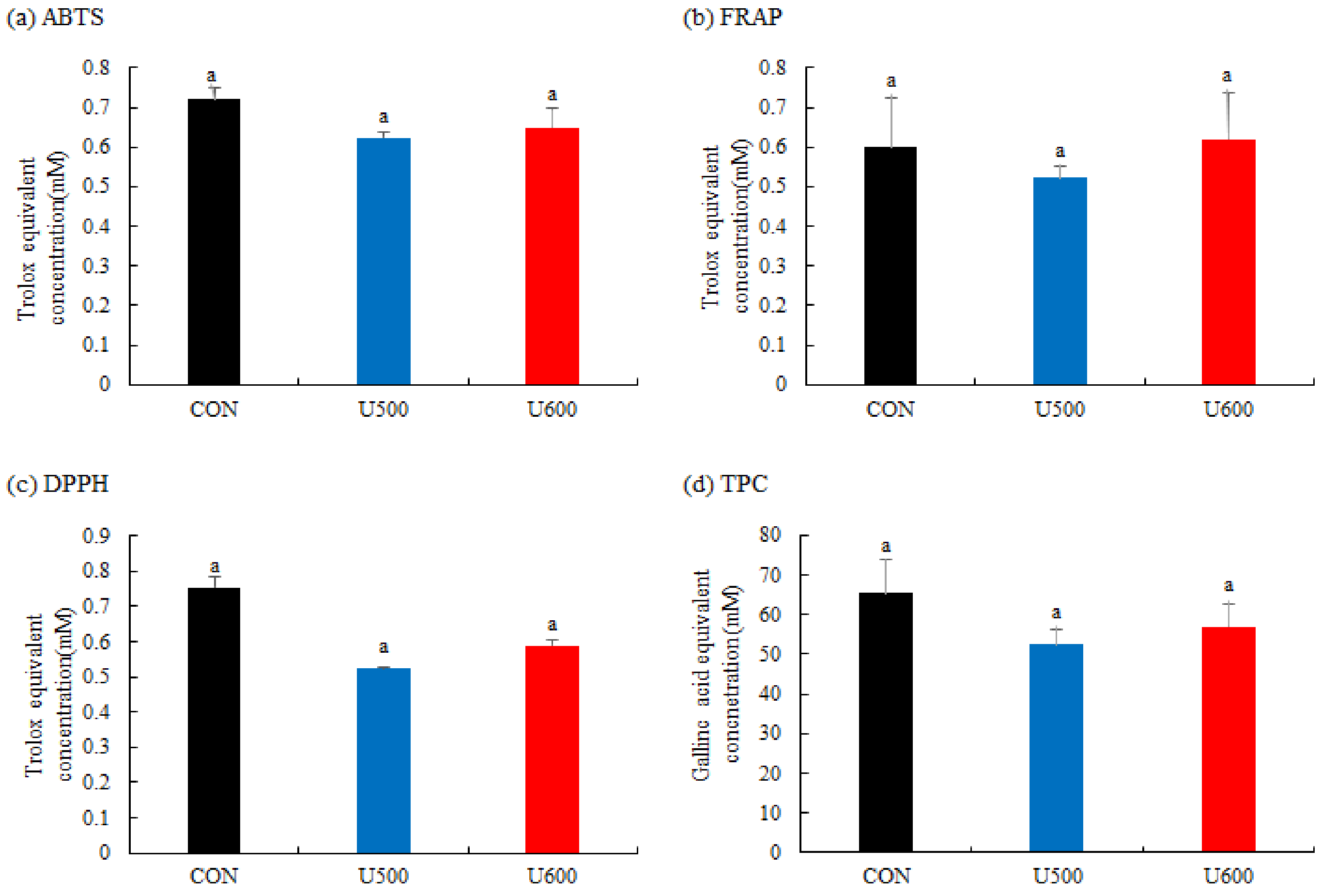

Figure S4. Antioxidant activity assay (a) ABTS; (b) FRAP; (c) DPPH; and (d) total phenolic contents (TPC) of GBP obtained after different UHP processing treatments. Values are expressed as the average of three biological replicates. Results shown in the bar graphs denoted by the same letter indicate absence of statistical difference, according to Duncan's multiple range test ($p < 0.05$).

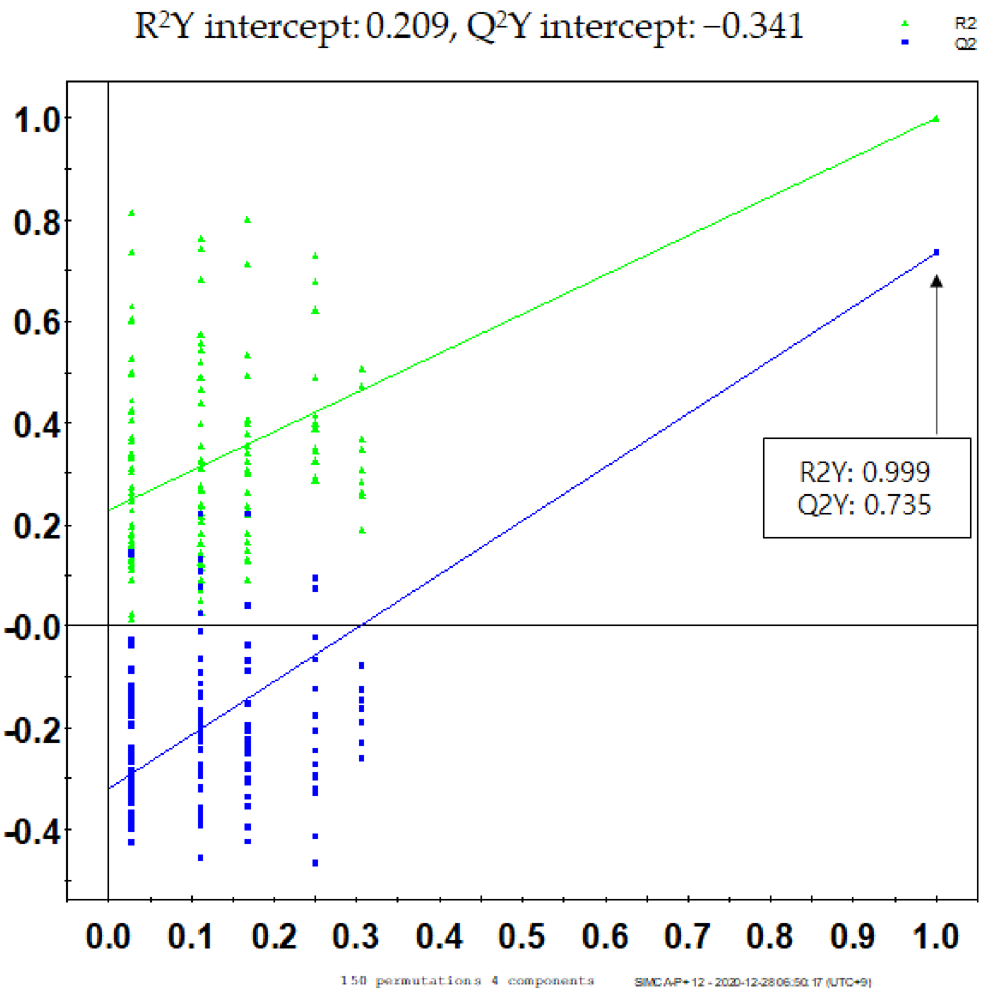

Figure S5. Validation plots of different solvent fractions of GBP analyzed by UHPLC-LTQ-Orbitrap-MS/MS. The Y axis of validation plot represent R^2Y (green colored triangle) and Q^2Y (blue colored squared) for every model, and the X axis indicates the correlation coefficient between original and permuted response data. The Y intercepts of plot for the R^2Y and Q^2Y in each model were represented in number.

Table S5. Differential metabolites in different solvent fractions of the GBP obtained after UHPLC-LTQ-Orbitrap-MS/MS analyses

Classification	Tentative metabolites[a]	RT (min)[b]	$[M-H]^-$	$[M+H]^+$	MW[c]	Molecular fomula	Delta ppm	Fragment pattern	Ref.[d]
Phenolic acids	Quinic acid	0.86	191.0569	193.0636	192	C7H12O6	2.1	191>85>57	[3]
	Gentisic acid	1.84	153.0195	155.0341	154	C7H6O4	0.4	153>109	[1]
	Protocatechualdehyde	3.07	137.0245	139.0393	138	C7H6O3	0.7	137>92	[9]
	Chlorogenic acid	3.85	353.0883	377.0848[f]	354	C16H18O9	-0.1	353>191>127>99	[2]
	Caffeic acid	4.02	179.0351	181.0497	180	C9H8O4	0.4	179>135>107, 91	[5]
	4-p-Coumaroylquinic acid	4.55	337.0937	339.108	338	C16H18O8	2	337>191>127>85	[10]
	5-Feruloylquinic acid	4.58	367.1036	369.1182	368	C17H20O9	0.7	367>191>127>84	[11]
	p-Coumaric acid	4.72	163.0406	165.0548	164	C9H7O3	3.2	163>119	[12]
Flavonoid	Quercetin	4.93	301.0359	303.0507	302	C15H10O7	1.7	301>273>245>150	[4]
	Kaempferol-glucoside	5.1	447.0932	449.1082	448	C21H20O11	-0.1	447>284>255	[4]
	Kaempferol	6.5	285.0402	287.0547	286	C15H10O6	-0.1	285>197>167	[4]
Lignan	7-Hydroxysecoisolariciresinol	4.98	377.1616	401.1572[f]	378	C20H26O7	2.9	377>347, 329>314	[13]
	Syringaresinol	5.1	417.1559	419.1711	418	C22H26O8	1.1	417>381>249	webDB
Fatty acid	Trihydroxy-octadecenoic acid	6.6	329.2325	353.2296[f]	330	C18H34O5	-0.5	329>229>211>183	[10]
	Oxo-dihydroxy-octadecenoic acid	6.89	327.2181	351.2143[f]	328	C18H32O5	1.7	327>309>291>273, 247	[14]
Ginsenoside	Ginsenoside Rb3	5.27	1123.592[g]	1101.582[f]	1078	C53H90O22	0.9	1123>1077>945>783, 637, 475	[8]
	Notoginsenoside R1	5.28	977.5334[g]	955.5231[f]	932	C47H80O18	0.4	977>931>637>475	[5]
	Psuedoginsenoside F11	5.4	799.4837	801.4995	800	C42H72O14	-1.6	799>653>491>391	[5]
	Ginsenoside Re	5.47	991.5461[g]	969.5385[f]	946	C48H82O18	-1.8	991>945>783, 637, 475	[5]
	Ginsenoside Rg1	5.51	845.4900[g]	823.4814[f]	800	C42H72O14	-0.5	845>799>491>391	[5]
	Malonylginsenoside Re	5.67	1031.543	1055.54[f]	1032	C51H84O21	-1.3	1031>987>945, 799	[6]
	Ginsenoside Rf	6.22	845.4909[g]	801.4996	800	C42H72O14	1	845>799>653>491, 415	[5]
	Malonyl-ginsenoside Rb1	6.31	1193.595	1195.615	1194	C57H94O26	-2.1	1193>1149>1107>945, 783, 621, 459	[7]
	Notoginsenoside R2	6.4	815.4789[g]	793.4697[f]	770	C41H70O13	-0.2	815>769>637, 475	[7]
	Ginsenoside Rb2/Rc	6.45	1077.581	1101.58[f]	1078	C53H90O22	-2.2	1077>945>783, 621	[5]
	Ginsenoside Rg2	6.51	829.4942[g]	807.4861[f]	784	C42H72O13	-1.5	829>784>475>391	[5]
	Ginsenoside Rd	6.68	991.5474[g]	969.5388[f]	946	C48H82O18	-1.8	991>945>783, 621	[5]
	Malonyl-ginsenoside Rd	6.74	1031.542	1055.539[f]	1032	C51H84O21	-1.2	1031>987>945>783, 621, 459	[5]
	Ginsenoside Rh1	6.76	683.437[g]	661.4277[f]	638	C36H62O9	-2.4	683>637>475>391	[5]
	Compound Mc1/Mx1/O	7.05	961.5362[g]	939.5281[f]	916	C47H80O17	-2.1	961>915>621>459	STD[e]
	Ginsenoside F1	7.27	683.438[g]	661.4296[f]	638	C36H62O9	0.3	683>637>475	[5]
	Ginsenoside Rg4	7.39	811.4847[g]	789.4777[f]	766	C42H70O12	-0.2	811>765>619	STD[e]
	Ginsenoside F4	7.4	765.4782	789.4772[f]	766	C42H70O12	-1.9	765>619>527	[8]
	Ginsenoside F2	7.4	829.4942[g]	807.4872[f]	784	C42H72O13	-2	829>783>621>537	STD[e]
	Ginsenoside Rh4	7.62	665.4261[g]	643.4181[f]	620	C36H60O8	0	665>619>561>471, 353	STD[e]
	Ginsenoside LXXV/Ginsenoside Rg3	7.75	829.4948	853.4958[f]	830	C42H72O13	0	829>783>621>537, 459	[8]
	Protopanaxatriol-20	8.47	521.3846[g]	499.3747[f]	476	C30H52O4	-0.2	521>475>391>373, 355	[8]
	Ginsenoside Rg5	8.56	765.4781	789.477[f]	766	C42H70O12	-1.7	765>603>441	[8]
	Ginsenoside Rh2	8.84	667.4418[g]	623.451	622	C36H62O8	-1.4	667>621>459>375	[8]
	Ginsenoside Rk2/Rh3	9.76	649.4312[g]	605.4417	604	C36H60O7	-0.2	649>603>457>373	[8]

[a] Different metabolites were identified from the variable important in projection (VIP) analysis with a cut-off value of 0.7 and *p*-value < 0.05. [b] Retention time. [c] Molecular weight. [d] References. [e] Standard of ginsenoside. [f] $[M+Na]^+$. [g] $[M+FA-H]^-$.

Supplementary references

1. Wang, L.; Halquist, M.S.; Sweet, D.H. Simultaneous determination of gallic acid and gentisic acid in organic anion transporter expressing cells by liquid chromatography-tandem mass spectrometry. *Journal of Chromatography B* **2013**, *937*, 91-96.
2. Jaiswal, R.; Müller, H.; Müller, A.; Karar, M.G.E.; Kuhnert, N. Identification and characterization of chlorogenic acids, chlorogenic acid glycosides and flavonoids from Lonicera henryi L.(Caprifoliaceae) leaves by LC-MS^n. *Phytochemistry* **2014**, *108*, 252-263.
3. Mata, A.; Ferreira, J.; Semedo, C.; Serra, T.; Duarte, C.; Bronze, M. Contribution to the characterization of Opuntia spp. juices by LC-DAD-ESI-MS/MS. *Food Chemistry* **2016**, *210*, 558-565.
4. Suh, D.H.; Jung, E.S.; Lee, G.M.; Lee, C.H. Distinguishing six edible berries based on metabolic pathway and bioactivity correlations by non-targeted metabolite profiling. *Frontiers in Plant Science* **2018**, *9*, 1462.
5. Mao, Q.; Bai, M.; Xu, J.-D.; Kong, M.; Zhu, L.-Y.; Zhu, H.; Wang, Q.; Li, S.-L. Discrimination of leaves of Panax ginseng and P. quinquefolius by ultra high performance liquid chromatography quadrupole/time-of-flight mass spectrometry based metabolomics approach. *Journal of Pharmaceutical and Biomedical Analysis* **2014**, *97*, 129-140.
6. Lee, M.Y.; Seo, H.S.; Singh, D.; Lee, S.J.; Lee, C.H. Unraveling dynamic metabolomes underlying different maturation stages of berries harvested from Panax ginseng. *Journal of ginseng research* **2020**, *44*, 413-423.
7. Lee, J.W.; Choi, B.-R.; Kim, Y.-C.; Choi, D.J.; Lee, Y.-S.; Kim, G.-S.; Baek, N.-I.; Kim, S.-Y.; Lee, D.Y. Comprehensive profiling and quantification of ginsenosides in the root, stem, leaf, and berry of Panax ginseng by UPLC-QTOF/MS. *Molecules* **2017**, *22*, 2147.
8. Wu, W.; Sun, L.; Zhang, Z.; Guo, Y.; Liu, S. Profiling and multivariate statistical analysis of Panax ginseng based on ultra-high-performance liquid chromatography coupled with quadrupole-time-of-flight mass spectrometry. *Journal of pharmaceutical and biomedical analysis* **2015**, *107*, 141-150.
9. Zeng, G.; Xiao, H.; Liu, J.; Liang, X. Identification of phenolic constituents in Radix Salvia miltiorrhizae by liquid chromatography/electrospray ionization mass spectrometry. *Rapid Communications in Mass Spectrometry* **2006**, *20*, 499-506.
10. Zengin, G.; Llorent-Martínez, E.; Sinan, K.I.; Yıldıztugay, E.; Picot-Allain, C.; Mahomoodally, M.F. Chemical profiling of Centaurea bornmuelleri Hausskn. aerial parts by HPLC-MS/MS and their pharmaceutical effects: From nature to novel perspectives. *Journal of pharmaceutical and biomedical analysis* **2019**, *174*, 406-413.
11. Keser, D.; Guclu, G.; Kelebek, H.; Keskin, M.; Soysal, Y.; Sekerli, Y.E.; Arslan, A.; Selli, S. Characterization of aroma and phenolic composition of carrot (*Daucus carota* 'Nantes') powders obtained from intermittent microwave drying using GC-MS and LC-MS/MS. *Food and Bioproducts Processing* **2020**, *119*, 350-359.
12. Jaitz, L.; Siegl, K.; Eder, R.; Rak, G.; Abranko, L.; Koellensperger, G.; Hann, S. LC-MS/MS analysis of phenols for classification of red wine according to geographic origin, grape variety and vintage. *Food Chemistry* **2010**, *122*, 366-372.
13. Eklund, P.C.; Backman, M.J.; Kronberg, L.; Smeds, A.I.; Sjöholm, R.E. Identification of lignans by liquid chromatography-electrospray ionization ion-trap mass spectrometry. *Journal of mass spectrometry* **2008**, *43*, 97-107.
14. Islam, A.K.M.M.; Hong, S.-M.; Lee, H.-S.; Moon, B.-C.; Kim, D.; Kwon, H. Identification and characterization of matrix components in spinach during QuEChERS sample preparation for pesticide residue analysis by LC?"ESI?"MS/MS, GC?"MS and UPLC-DAD. *Journal of food science and technology* **2018**, *55*, 3930-3938.
